# FIRST-YEAR OCCUPATIONAL THERAPY STUDENTS KNOWLEDGE OF AGING; SUGGESTIONS FOR CURRICULUM REQUIREMENTS

**DOI:** 10.1093/geroni/igac059.208

**Published:** 2022-12-20

**Authors:** LaVona Traywick, Brittany Saviers, Terry Griffin, Teressa Brown

**Affiliations:** Arkansas Colleges of Health Education, Fort Smith, Arkansas, United States; University of Central Arkansas, Conway, Arkansas, United States; Kansas State University, Manhattan, Kansas, United States; Arkansas Colleges of Health Education, Fort Smith, Arkansas, United States

## Abstract

There is a rising shortage of allied healthcare professionals, including occupational therapists, to meet the current and expected needs of the senior adult population. To educate occupational therapy students, there are national standards that all programs have to meet. Inconsistency occurs because there is not a set national curriculum. It is assumed that students will enter their respective occupational therapy programs with a base knowledge of aging due to prerequisite requirements. With IRB approval, that assumption was measured over four consecutive years at one prominent OT program. 192 first-semester occupational therapy students were administered the Facts on Aging Quiz along with additional questions regarding birth year and anticipated employment. Results showed that first-year occupational therapy students’ knowledge of aging was poor (67.9% mean) regardless of their age or population work preference. Only 11.5% stated geriatrics was their preferred population with which to work; most students stated pediatrics. Statistical tests indicated a trend of decreasing mean scores of the cohorts. If this trend of decreasing gerontological literacy exists in occupational therapy, other health care disciplines may be experiencing similar fates. It appears there is a considerable gap between the needs of society and the knowledge base/desired population work preference of those entering healthcare education. Strategies to address that gap need to be addressed to prepare occupational therapy students and other health care practitioners to best meet the needs of the current population. Based on these study results, more emphasis needs to be placed on gerontological literacy for new occupational therapy students.

